# Time lapse: A glimpse into prehistoric genomics

**DOI:** 10.1016/j.ejmg.2019.03.004

**Published:** 2020-02

**Authors:** Darren K. Griffin, Denis M. Larkin, Rebecca E. O'Connor

**Affiliations:** a School of Biosciences, University of Kent, Canterbury, CT2 7NJ, UK; b Department of Comparative Biomedical Sciences, Royal Veterinary College, University of London, London, NW1 0TU, UK

**Keywords:** Dinosaur, Chromosome, Karyotype, Comparative, Genome evolution

## Abstract

For the purpose of this review, ‘time-lapse’ refers to the reconstruction of ancestral (in this case dinosaur) karyotypes using genome assemblies of extant species. Such reconstructions are only usually possible when genomes are assembled to ‘chromosome level’ i.e. a complete representation of all the sequences, correctly ordered contiguously on each of the chromosomes. Recent paleontological evidence is very clear that birds are living dinosaurs, the latest example of dinosaurs emerging from a catastrophic extinction event. Non-avian dinosaurs (ever present in the public imagination through art, and broadcast media) emerged some 240 million years ago and have displayed incredible phenotypic diversity. Here we report on our recent studies to infer the overall karyotype of the Theropod dinosaur lineage from extant avian chromosome level genome assemblies. Our work first focused on determining the likely karyotype of the avian ancestor (most likely a chicken-sized, two-legged, feathered, land dinosaur from the Jurassic period) finding karyotypic similarity to the chicken. We then took the work further to determine the likely karyotype of the bird-lizard ancestor and the chromosomal changes (chiefly translocations and inversions) that occurred between then and modern birds. A combination of bioinformatics and cross-species fluorescence *in situ* hybridization (zoo-FISH) uncovered a considerable number of translocations and fissions from a ‘lizard-like’ genome structure of 2n = 36–46 to one similar to that of soft-shelled turtles (2n = 66) from 275 to 255 million years ago (mya). Remarkable karyotypic similarities between some soft-shelled turtles and chicken suggests that there were few translocations from the bird-turtle ancestor (plus ∼7 fissions) through the dawn of the dinosaurs and pterosaurs, through the theropod linage and on to most to modern birds. In other words, an avian-like karyotype was in place about 240mya when the dinosaurs and pterosaurs first emerged. We mapped 49 chromosome inversions from then to the present day, uncovering some gene ontology enrichment in evolutionary breakpoint regions. This avian-like karyotype with its many (micro)chromosomes provides the basis for variation (the driver of natural selection) through increased random segregation and recombination. It may therefore contribute to the ability of dinosaurs to survive multiple extinction events, emerging each time as speciose and diverse.

## 1 Foreword

Being asked to give a presentation about dinosaur genomes (in a conference that is fundamentally about preimplantation and prenatal diagnosis) presented somewhat of a challenge. Firstly, a title; ‘Time lapse’ - to most of the audience - conjures up time-lapse imaging of human embryos; the title that you see above was thus deliberately mischievous as the talk had nothing to do with this. Second, how, scientifically, does our work link to the rest of the conference? The point here is that techniques such as array CGH, NGS and karyomapping would not be possible for chromosome screening unless the human genome was assembled to ‘chromosome-level’; that is a genome with all the sequences assigned to their rightful place on the chromosome.

## 2 Chromosome-level assemblies

Genomics needs cytogenetics. In order to navigate around any complex structure, a map is essential and a genome is no exception (paraphrasing Lewin et al., 2009). Nature provides us with the basis of a genomic map in the form of a karyotype. More commonly thought of as a means of detecting disease in humans (Down Syndrome is the best-known example), the karyotype of an individual species is the most fundamental low-resolution genomic map available. Indeed, the ultimate aim of any genome assembly is each sequence assigned to a specific locus on a chromosome, and thus a complete representation of all the sequences, correctly ordered contiguously on each of the chromosomes. When whole genome sequences fall short of this chromosome-level assembly (this is true of the majority of sequenced genomes), their use for critical aspects of evolutionary and applied biology is limited. As an example, chromosome-level assemblies have been essential for agricultural species because an established order of DNA markers is a pre-requisite for the establishment of phenotype-to-genotype associations for gene-assisted selection. When this was achieved for cattle, sheep, pig and chicken, high-resolution SNP genotyping became effective for genomic association studies. This in turn facilitated mapping of Mendelian disorders, accurate identification of overt and cryptic chromosome translocations, discovery of quantitative trait nucleotides (QTNs) and expression quantitative trait loci (eQTLs) and the study of long-range regulatory interactions. Such studies ultimately lead to increased efficiency in food production and improved global food security. Once such assemblies are built for numerous species, comparative genomics becomes possible *in silico* and identification of chromosome rearrangements not easily detected by basic karyotyping (e.g. cryptic translocations) is achievable by molecular cytogenetics. Chromosome-level assemblies are also essential to address basic biological questions related to genome evolution e.g. the reasons why chromosomes break and re-form (and why sometimes they don't) as well as for understanding the significance and genomic correlates of chromosomal breakpoint regions and the reasons why blocks of genes (homologous synteny blocks) are maintained together during evolution. Far more than simply a descriptive science therefore, cytogenetics provides a backbone for the visualization of any genome, a means through which we can understand the relationship between genome and phenome more fully and a route to comparative genomics from a whole genome perspective. Comparative genomics, in turn, permits the establishment of overall genome structure of less well described species (by comparison to those better described) and the mapping of gross genomic changes that led to each species' characteristic karyotype. The purpose of this review is to summarise how we studied chromosome-level assemblies of bird species and thereby provided novel insight into the karyotypes of the avian forebears - the Theropod dinosaurs.

## 3 What are dinosaurs?

The first point to make is that it is technically incorrect to state either that birds *evolved from* dinosaurs, or that they are *related to* dinosaurs. More correctly, the latest paleontological evidence is very clear that *birds are dinosaurs.* We are all aware the effect that dinosaurs have had on popular culture and the creative arts since the very first fossil discoveries. This is aided, in no small part, via film, television, press, art and literature. Indeed, rather than being a group of animals that were wiped out by the K-Pg extinction event caused by the Chicxulub meteor, Dinosaurs are in fact the survivors of several extinction events. In a recent study, using bioinformatics and molecular cytogenetics, we were able to provide evidence suggesting that this longevity and resilience may be due, in part, to their unique genomic structure, i.e. their karyotype.

Around 325 mya (million years ago), amniotes diverged into Synapsids - the lineage that ultimately became mammals and Diapsids - the reptile/bird lineage. There are ∼17,500 extant diapsid species, ∼10,500 of which are birds. Crocodilians, dinosaurs, pterosaurs, turtles and birds all share a common ancestor that lived 275 mya (Shedlock and Edwards, 2009; Hedges et al., 2015), with the turtles (testudines) diverging first (around 255 mya), the crocodilians around 252 mya, the forebears of the Pterosaurs ∼245 mya and the first dinosaurs appearing ∼240 mya. Dinosaurs are formally defined as “the clade including *Triceratops*, *Passer* (songbirds) and all of the descendants of their common ancestor”. For the first 30 million years of their evolution (until around 210 mya) there were relatively few dinosaur species, but by the mid Jurassic period, the number of species, their geographical spread and their body size had all increased significantly (Benton et al., 2014). The next 135 million years of dinosaur evolution is remarkable for being a period not only for when dinosaurs were the dominant vertebrates but also for being a time when they displayed a remarkable range of species diversity. Dinosaurs survived several extinction events including the Carnian-Norian (CNEE) 228 mya and the End-Triassic mass extinction event (ETME) 201 mya that also devastated the crocodilian ancestors (leaving only 23 living) species. There are now over 1000 known species of dinosaur (excluding birds) in the fossil record with around 30 more appearing each year (Weishampel, 2004). Despite the number and diversity of dinosaurs being devastated by the Cretaceous-Paleogene (K-Pg) extinction event 66 mya, survivors of this event emerged as modern birds, with over 10,500 species of all shapes and sizes. In understanding this group of animals, genomic and cytogenetic studies of extant birds can be a useful adjunct to paleontology, due to the inherent difficulties in fossil dating. Either way, the dinosaur ancestor of birds is generally considered to be a bipedal, terrestrial, relatively small Jurassic dinosaur with limited flying ability, not dissimilar to land fowl such as chicken or quail (Witmer, 2002).

## 4 Avian genomics

Until the publication in 2014 of a revised avian phylogeny, based on genomic data, the timing of avian diversification has been a subject of much debate (Jarvis et al., 2014). The first avian divergence is considered to have occurred about 100 mya when the Paleognathae (Ratites and Tinamous) diverged from the Neognathae (Galloanseres and Neoaves which subsequently diverged ∼80 mya). The Galloansere divergence into the *Galliformes* (landfowl e.g. chicken) and *Anseriformes* (waterfowl e.g. ducks) occurred around the time of the K-Pg extinction event (see below). The major divergences of the Neoaves into Columbea (e.g. pigeons) and Passarea (e.g. songbirds) are now dated to before the K-Pg boundary (67–69 mya). Data from the Jarvis et al. analysis and Prum et al., (2015) suggests that following the mass extinction event thought to be caused by the Chicxulub meteor strike (Schulte et al., 2010), there was a period of rapid avian speciation, with 36 lineages appearing over the relatively short period of 10–15 million years (Jarvis et al., 2014). Genomic studies have therefore, updated our understanding of dinosaur genomics and its relationship to phenotype and diversity (Zhang et al., 2014a; Jarvis et al., 2014). The overall genomic structure (i.e. karyotype) of dinosaurs was something that had until now, been understudied and was therefore the subject of our investigations.

## 5 Karyotypic evolution in the dinosaurs

In the absence of cellular material (or even relatively intact DNA) data from genome sequence assemblies of living species provide us with the ability to reconstruct karyotypic structures of extinct lineages by inference. We can do this on the proviso that genomes are assembled at, or close to, chromosome-level (see above). In a study that coincided with the publication of the multiple avian genomics and phylogeny papers in (Zhang et al., 2014b; Jarvis et al., 2014) we analysed (near) chromosome-level assemblies from six living birds. Using an *Anolis* lizard outgroup we inferred the most likely ancestral karyotype of all birds. We then went on to reconstruct the most likely sequence of events that led to contemporary karyotypes in birds. We provided evidence that the chicken (*Gallus*) was the closest karyotypically to the reconstructed ancestral pattern, with budgerigar (*Melopsittacus undulatus)* and zebra finch (*Taeniopygia guttata)* experiencing the greatest number of inter- and intra-chromosomal rearrangements respectively (Romanov et al., 2014). More recently, we returned to the reconstruction of the ancestral karyotype using an algorithmic approach applied to fragmented genome assemblies. In that study (Damas et al., 2018) we made use of the DESCHRAMBLER algorithm to perform large-scale analysis of ancestral avian chromosome structure in 14 key nodes of avian evolution. This permitted analysis from the avian ancestor to the ancestor of the Estrildidae, Thraupidae and Fringillidae families. Our results provided critical insight into the variability of rearrangement rates during avian evolution, permitting the detection of patterns related to the chromosome distribution of evolutionary breakpoint regions (EBRs) and of microchromosomes.

**Fig. 1:**
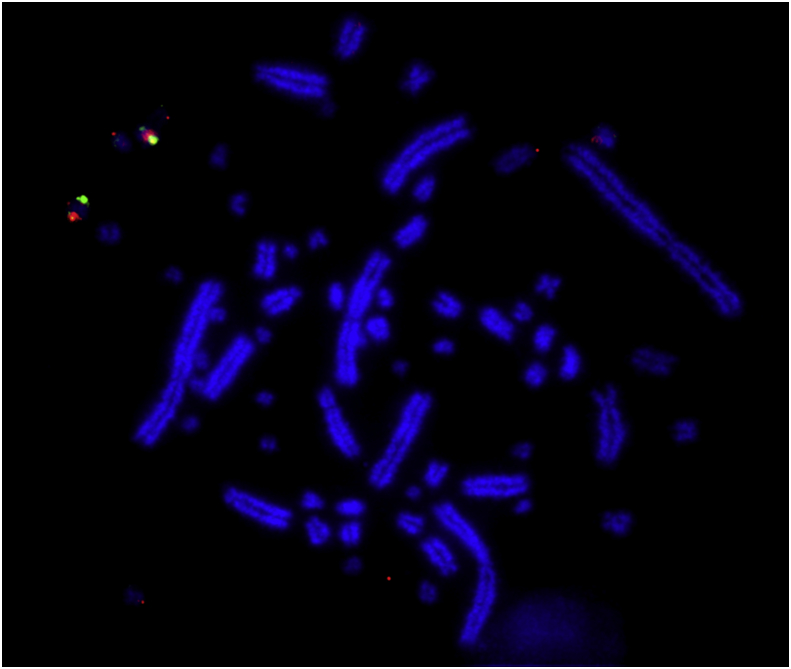
Hybridization of red and green fluorescent probes derived from chicken microchromosome 27 to the metaphases of *Apalone spinifera* (spiny soft-shelled turtle). The results show homology with a single microchromosome in the turtle species. (For interpretation of the references to colour in this figure legend, the reader is referred to the Web version of this article.)

Last year (O'Connor et al., 2018c) we applied a comparable approach to recreate the most likely ancestral karyotype of diapsids. Using a combination of a bioinformatics and molecular cytogenetics we developed a FISH (BAC) probe set that would hybridise directly across species that diverged hundreds of millions of years ago (Damas et al., 2017). The BACs used gave strong hybridization signals to turtle (Fig. 1) and some *Anolis carolinensis* (lizard) chromosomes and those of two turtles *Trachemys scripta* (red earned slider) and *Apalone spinifera* (spiny soft-shelled turtle). Although these two turtles do not have chromosome-level assemblies, molecular cytogenetic analysis allowed us to anchor the series of events from the perspective of a bird-turtle ancestor*.* A combination of this molecular cytogenetic approach and bioinformatics allowed us to recreate the inter- and intrachromomsomal changes that occurred from the ancestral diapsid ancestor, to the archelosaur (bird-turtle) ancestor (Benton et al., 2015), through the theropod dinosaur lineage to modern birds.

Our data, and interpretations from it, provide substantial evidence that many of the features associated with a ‘typical avian-karyotype’ were established prior to the turtle divergence 255 mya indicating that most chicken (and by inference, ancestral avian) chromosomes 1–28 + Z are syntenic to those of the spiny soft-shelled turtle *Apalone spinifera* (2*n* = 66). Hybridization of some probes to the chromosomes of *Trachemys scripta* (2*n* = 50) and *Anolis carolinensis* metaphases (2*n* = 36) revealed some chromosomes with microchromosomal homologues attached suggesting either a fusion to macrochromosomes or, more likely, retention of the ancestral state present in the diapsid ancestor. Our results therefore suggest that the ‘avian-like pattern’ was in place around 255 mya. Subsequent work by our group using these probes on the chromosomes of 22 avian species across 10 orders also revealed that these microchromosomes have since remained unchanged across the majority of avian species (O'Connor et al., 2018b).

**Fig. 2:**
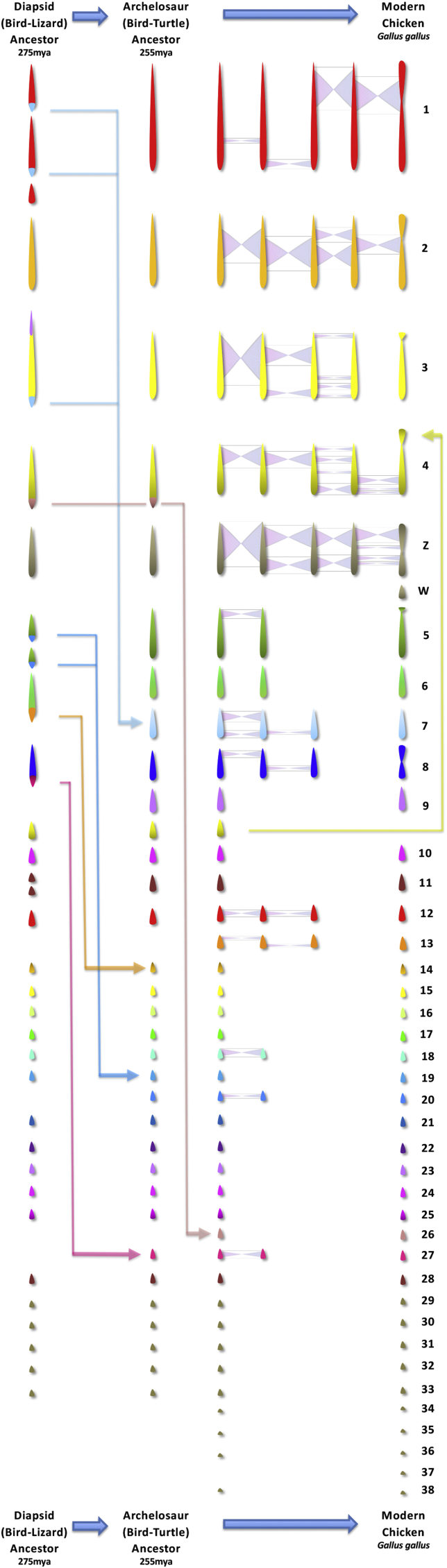
Karyotype evolution from the diapsid ancestor, via the theropod dinosaur lineage, to modern birds. The basic “avian” pattern was present about the time the dinosaurs emerged 240 mya. Thereafter, mostly chromosome inversions were the mechanisms of change.

A picture then emerges (see Fig. 2) of a diapsid ancestral karyotype (∼275 mya) with a chromosome number of 2n = 36–46 - roughly half would have been macro- and half microchromosomes (Beçak et al., 1964; Alföldi et al., 2011). Rapid rearrangement over about 20 million years to a pattern similar to *Apalone spinifera* appears to have then occurred. These conclusions are consistent with previous studies using chicken macrochromosome paints on Chinese soft-shelled turtle (*Pelodiscus sinensis*) (2n = 66) (Matsuda et al., 2005), *Trachemys scripta* (Kasai et al., 2012) and the painted turtle (*Chrysemys picta*) chromosomes (both 2*n* = 50) (Badenhorst et al., 2015) which provide evidence that turtle and bird macrochromosomes are precise counterparts of one another. Since 255 mya only ∼7 fissions are required to form the pattern that we see in Ratites, Galliformes, Anseriformes, Columbaea and Passeriformes (among other birds). Determining how and when these changes occurred is difficult, however if a similar rate of fission that occurred from 275 to 255 mya carried on for another 15 million years, a complete bird-like karyotype would have emerged before the appearance of the earliest dinosaurs and pterosaurs (Baron et al., 2017). At the other extreme, a complete cessation of fission events 255 mya would indicate that the earliest dinosaur and pterosaur karyotypes were more similar to that of *Apalone spinifera or Pelodiscus sinensis*. David Burt, (2002) suggested that most avian microchromosomes were present in the avian ancestor >80 mya (Cracraft et al., 2015), suggesting that it probably had a karyotype of around 2*n* = 60. Our recent data supports the idea that this karyotype was in place long before and likely came before any reduction in genome size (O'Connor et al., 2018c). Indeed Uno and colleagues (Uno et al., 2012) suggested that the archelosaur ancestor probably had microchromosomes like turtles. There is however, evidence of an association between genomes with fewer chromosomes (and no microchromosomes) and larger genome sizes around 2.5–3 Gb, as seen in most mammals (Kapusta et al., 2017) and crocodilians (St John et al., 2012). Repetitive elements provide substrates for interchromosomal rearrangement, commonly seen in mammals but rare in birds, suggesting that the avian karyotype provides fewer opportunities for interchromosomal rearrangement due to a lack of recombination hotspots (despite a higher overall recombination rate) (Kawakami et al., 2014; Smeds et al., 2016), repeat structures (Mason et al., 2016; Warren et al., 2017; Gao et al., 2017), and endogenous retroviruses (Romanov et al., 2014; Cui et al., 2014; Farré et al., 2016). Intrachromosomal rearrangements and fusions are not however impeded in this model. The evidence therefore suggests that the avian-like karyotype was in place first, followed by a reduction in genome size, followed by flight. Also, recently we established that there is purifying selection acting on at least several smaller chromosomes (Damas et al., 2018). These are depicted in Fig. 2.

## 6 Chromosome inversion and the role of gene ontology analysis

Aside from ∼7 fissions proposed in this model, the primary mechanism for chromosomal rearrangement in the avian ancestor after 255 mya was likely to be via chromosomal inversion (also depicted in Fig. 2). Using ancestral genome reconstruction tools ((Multiple Genome Rearrangement and Analysis MGRA - (Avdeyev et al., 2016)), we generated 19 contiguous ancestral regions (CARs). These CARs likely represented the chromosomes of the diapsid ancestor and when compared to the genomes of living birds, resulted in the identification of rearrangements between the diapsid ancestor and chicken genomes. Through this approach we were able to identify 49 chromosome inversions (although it is likely to be an underestimate due to the variation in sequence coverage, particularly on the smallest bird microchromosomes). Rates of change are difficult to establish but there is some evidence of intrachromosomal change speeding up in modern birds, even in the chicken, which is thought to be very similar chromosomally to the avian common ancestor (Romanov et al., 2014). A higher degree of intrachromosomal change has been reported in some avian groups, with several studies suggesting that higher rates occur within the songbirds (Skinner and Griffin, 2011; Zhang et al., 2014b; Farré et al., 2016), the most speciose group. Bursts of speciation may therefore have also been accompanied by increased rates of chromosome inversion in other dinosaur groups.

In our most recent study of dinosaur karyotypes (O'Connor et al., 2018c), we identified nearly 400 HSBs (homologous synteny blocks) – chromosome regions that tend to stay together during evolution, delineated by EBRs (evolutionary breakpoint regions). Previous genomic studies in other species have found that EBRs are often located within gene-dense loci, with genes related to lineage-specific biology, transposable elements and other repetitive sequences (Nadeau and Taylor, 1984; Pevzner and Tesler, 2003; Hillier et al., 2004; Rao et al., 2012). Sequences that stay together during evolution (HSBs) however have a higher degree of developmental genes and regulatory elements (Larkin et al., 2009; Warren et al., 2017). Random breakage during karyotype evolution is of course a possibility (Nadeau and Taylor, 1984) however, there is mounting evidence that the larger HSBs and selected EBRs are maintained non-randomly (Larkin et al., 2003; Pevzner and Tesler, 2003; Farré et al., 2016). Regions more prone to breakage (such as recombination hotspots or open chromatin areas), and chromosome breaks that do not disturb key genes or provide a selective advantage, are more likely to be fixed in populations (Farré et al., 2016). In other words, chromosome rearrangement may serve a functional purpose.

Analysis of HSBs in the 2018 study (O'Connor et al., 2018c) using GO (gene ontology) tools, revealed significant enrichments relevant to amino acid transmembrane transport and signalling as well as synapse/neurotransmitter transport, nucleoside metabolism, cell morphogenesis and cytoskeleton, and sensory organ development. Previous studies have suggested that HSBs are enriched for GO terms related to phenotypic features that remain constant (Larkin et al., 2009). These results are therefore consistent with this hypothesis. The EBRs however, are often considered to be where the most change in genome evolution resides (Sankoff, 2009). Our previous work found GO terms in avian EBRs that were associated with specific adaptive features, e.g. enrichment for forebrain development in the budgerigar EBRs (consistent with vocal-learning) (Farré et al., 2016). In our most recent study however, we identified significant enrichments in genes and single GO terms relevant to chromatin modification and chromosome organization as well as proteasome/signalosome structure (O'Connor et al., 2018c).

## 7 How does the karyotype impact on the phenotype of dinosaurs?

This apparent lack of karyotypic rearrangement over a period of 255 million years suggests that this pattern of genome organization may contribute to the evolutionary success of this animal group. The large number of chromosomes, and the presence of microchromosomes with high recombination rates, may in fact lead to greater variation through increased genetic recombination and increased random chromosome segregation. Although the presence of multiple chromosomes is not the only means by which variation can be generated, it may indeed explain the apparent paradox of a group with incredible phenotypic diversity but very little interchromosomal change.

Our results suggest therefore that if we had the opportunity to make chromosome preparations from tissue of some of our favourite theropod dinosaurs (*Tyrannosaurus rex* and *Velociraptor* are both members of the group) then karyotype and zoo-FISH results would differ very little from that of a modern chicken, pigeon, duck or ostrich. While it is always possible that some groups underwent significant interchromosomal change, (kingfishers (Christidis, 1990) (many fissions), parrots (Nanda et al., 2007; O'Connor et al., 2018a) and falcons (Damas et al., 2017; Joseph et al., 2018) (many fusions) are modern examples of this.

The discovery that the avian karyotype likely dates back further than previously thought complements paleontological research that demonstrates that features such as feathers and pneumatised skeletons arose first among more ancient dinosaur or archosaurian ancestors (Zhou, 2004; Baron et al., 2017). Dinosaurs were the dominant group of animals for around two hundred million years, with significant radiations occurring in response to two mass extinction events and, despite being almost wiped out by a third (the K-Pg meteor impact), their resilience as a highly diverse and speciose clade (extant birds) (Barrowclough et al., 2016) is evident.

## 8 Conclusions

Investigating chromosomal changes that occurred during evolution has parallels with the analysis of clinical patient samples. Aneuploidy is rare, but, in our evolutionary studies, chromosome inversions, translocation, fissions, fusions, insertions and deletions all appear. The analysis of bioinformatic data generated through this research and the recreation of chromosomal diagrams is common to both. One rewarding aspects of the work however was exploring the phenotypic associations with the data. In recreating dinosaur karyotypes we were not just making inferred descriptions. Rather, we were tracing the gross genome organization and evolution of ancient chromosomes and making credible conclusions about how this might impact on phenotypic diversity, physiology, and evolutionary adaptation (Berv and Field, 2018). The press interest in the study was also phenomenal however the question “are you going to recreate Jurassic Park?” was the one that seemed to be the most asked. We want go there (but if you want to read our thoughts on this then please see https://theconversation.com/jurassic-world-can-we-really-resurrect-a-dinosaur-97383). A cameo in the next Jurassic World film however? Well, Mr Spielberg – if you're listening ….

## Funded

This research was funded by the Biotechnology and Biological Sciences Research Council [BB/K008226/1 and BB/J010170/1 to D.M.L, and BB/K008161/1 to D.K.G].
